# The role of metacaspase 1 in *Plasmodium berghei* development and apoptosis

**DOI:** 10.1016/j.molbiopara.2007.01.016

**Published:** 2007-05

**Authors:** Ludovic Le Chat, Robert E. Sinden, Johannes T. Dessens

**Affiliations:** a Department of Infectious and Tropical Diseases, London School of Hygiene & Tropical Medicine, Keppel Street, London WC1E 7HT, United Kingdom; b Division of Cell & Molecular Biology, Imperial College, London SW7 2AZ, United Kingdom

**Keywords:** *Plasmodium berghei*, Metacaspase, Apoptosis

## Abstract

The malaria parasite encodes a wide range of proteases necessary to facilitate its many developmental transitions in vertebrate and insect hosts. Amongst these is a predicted cysteine protease structurally related to caspases, named *Plasmodium* metacaspase 1 (PxMC1). We have generated *Plasmodium berghei* parasites in which the PbMC1coding sequence is removed and replaced with a green fluorescent reporter gene to investigate the expression of PbMC1, its contribution to parasite development, and its involvement in previously reported apoptosis-like cell death of *P. berghei* ookinetes. Our results show that the *pbmc1* gene is expressed in female gametocytes and all downstream mosquito stages including sporozoites, but not in asexual blood stages. We failed to detect an apparent loss-of-function phenotype, suggesting that PbMC1 constitutes a functionally redundant gene. We discuss these findings in the context of two other putative *Plasmodium* metacaspases that we describe here.

## 1 Introduction

Malaria remains the most important parasitic human disease responsible for millions of deaths each year. The life cycle of malaria parasites is complex involving multiple life stages and requiring two hosts: mosquito and human. The uptake of gametocytes by the mosquito leads to rapid gametogenesis and fertilisation. Ookinetes are formed in the mosquito midgut lumen, which cross the midgut wall and transform into oocysts. Each oocyst can produce thousands of sporozoites, which after release traverse the mosquito hemolymph and infect the salivary gland tissues before they can enter the human host via the mosquito bite. Following inoculation, sporozoites infect hepatocytes and develop into liver schizonts. Liver schizont produce merozoites, which upon release into the blood stream initiate the erythrocytic cycle, the prime cause of clinical symptoms in malaria patients.

Existing malaria control strategies are hampered by increasing parasite resistance in the field to established antimalarial drugs, as well as by widespread insecticide resistance in the mosquito vectors. Effective antimalarial vaccines are only at the preliminary stages of development. There is thus a great need to develop novel strategies for malaria control. Proteases are attractive antimalarial targets because of their indispensable roles in parasite development and infectivity. A wide variety of proteases of malaria parasites have been described [1]. Amongst these are cysteine proteases belonging to the Peptidase_C14 family (clan CD) which include caspases. Caspases belong to a distinct class of cysteine proteases with a so called Caspase Hemoglobinase Fold (CHF) [2]. Recently, two new families of predicted CHF proteases closely related to the caspases were identified, named paracaspases and metacaspases [3].

In metazoa, caspases are centrally involved with the molecular machinery of programmed cell death (apoptosis), and are responsible for many of the biochemical and morphological changes that accompany it. They cleave a variety of proteins ultimately leading to the disintegration of the cell [4]. Metacaspases have thus far only been identified in organisms that do not possess classic caspase genes: plants, fungi and the parasitic protists *Trypanosoma*, *Leishmania* and *Plasmodium*, and there is some evidence linking metacaspases to apoptosis in these organisms [5–10]. In *Plasmodium falciparum* a metacaspase gene (GenBank accession CAD52669, here named PfMC1) has been described possessing consensus histidine and cysteine residues that typically form the catalytic dyad in this family of proteases [1,3], suggesting that this parasite species may possess a mechanism of programmed cell death. Indeed, apoptosis-like DNA fragmentation has been reported to occur in blood stage parasites of *P. falciparum* in response to chloroquine treatment [11]. In addition, ookinetes of *Plasmodium berghei* were reported to undergo an apoptosis-like mechanism of cell death resulting in very substantial parasite losses [12]. In this study we investigate the role of PbMC1 in the reported programmed cell death in *Plasmodium* by constructing PbMC1-deficient parasites. We discuss our findings in the context of two additional *Plasmodium* metacaspase-like proteins that we describe here.

## 2 Materials and methods

### 2.1 Parasite maintenance, culture and purification

*P. berghei* ANKA (clone 2.34) parasites were maintained in female TO mice by mechanical blood passage and regular transmission in *Anopheles stephensi*, as described [13]. Ookinete cultures were set-up as described [12,13] both with and without prior removal of white blood cells by CF11 columns. In general, 21–24 h ookinete cultures were centrifuged at 1500 × *g* for 5 min and the cell pellet incubated on ice in 20 volumes of 0.17 M NH_4_Cl to lyse the red blood cells. In some experiments ookinetes were further purified on Nycodenz cushens. Finally, parasites were concentrated by centrifugation at 1500 × *g* for 5 min, resuspended in RPMI 1640 or phosphate buffered saline, and immediately analysed.

### 2.2 Generation of PbMC1-KO parasites

A 720 bp fragment was amplified by PCR from *P. berghei* genomic DNA with primers IF-MC3′-F (TAAACCATTGGTCATACCAAAAAATAATCAAAAAAATAACCAA) and IF-MC3′-R (CGGGCCGCTCTAGCATGACCAGGCTCAATAATTGAACA) and introduced into *Nde*I-digested pLP-DHFR2 via In-Fusion PCR cloning (BD Biosciences). The resulting plasmid, pLP-DHFR/MC, contains a *loxP*-prokaryotic promotor cassette (BD Biosciences), followed by a modified *Toxoplasma gondii* dihydrofolate reductase (*dhfr*) gene cassette [14], followed by the 3′UTR of *pbmc1*. A 680 bp fragment was amplified by PCR from *P. berghei* genomic DNA with primers pDNR-ΔMC-F (ACGAAGTTATCAGTCGACGGTACCCCATCATAAAGCAAAAAGC) and pDNR-ΔMC-R (ATGAGGGCCCCTAAGCTTATTTAACATAAATTTTGTCCATTT) and introduced into *Sal*I/*Hin*dIII-digested pDNR-EGFP via In-Fusion PCR cloning. The resulting plasmid, pDNR-ΔMC/EGFP, contains two *loxP* sites flanking the 5′UTR plus first 11 codons of *pbmc1* fused in-frame to the enhanced green fluorescent protein (*egfp*) gene, followed by the 3′UTR of *P. berghei dhfr*. The *pbmc1*-specific insert contained within pDNR-ΔMC/EGFP was introduced into pLP-DHFR/MC via Cre-*loxP* site-specific recombination (BD Biosciences) to give the transfection vector pLP-ΔMC/EGFP. Prior to performing transfections this plasmid was digested with *Kpn*I and *Not*I to remove the vector backbone. Parasite transfection, pyrimethamine selection and dilution cloning were performed as previously described [15]. Genomic DNA extraction, and Southern blot were performed as previously described [14].

### 2.3 Apoptosis assays

Phosphatidylserine (PS) translocation was studied with fluorescein isothiocyanate (FITC)-conjugated Annexin V in conjunction with propidium iodide, by using the Annexin-FITC Apoptosis Detection Kit (Sigma–Aldrich) according to manufacturer's instructions. DNA condensation in the nucleus was assessed using acridine orange staining as described [12]. DNA fragmentation was assessed by *in situ* terminal deoxynucleotidyl transferase mediated dUTP-biotin nick end labelling (TUNEL), by using the ApopTag^®^ Fluorescein In Situ Apoptosis Detection Kit (Chemicon International) according to manufacturer's instructions. Caspase activation was assessed with sulphorhodamine-VAD-FMK inhibitor of caspases, by using the CaspaTag™ Pan-Caspase In Situ Assay Kit (Chemicon International) according to manufacturer's instructions.

## 3 Results

### 3.1 Description of *Plasmodium* metacaspases

**Fig. 1:**
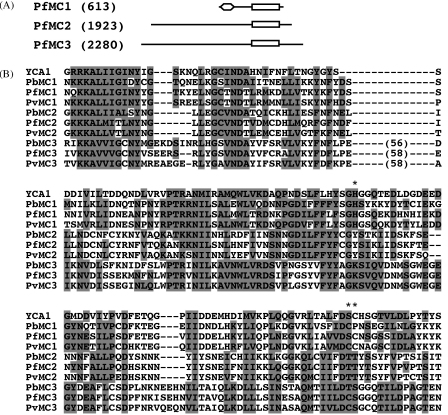
Structure of PxMC1, PxMC2 and PxMC3. (A) Schematic diagram of the three proteins in *P. falciparum* with their predicted sizes in amino acids shown in parentheses. The conserved domains are the Peptidase_C14/Caspase domain (rectangle) and the C2 domain (diamond). (B) ClustalW multiple alignment (default parameters) of the conserved part of the Peptidase_C14/Caspase domains of PxMC1, PxMC2 and PxMC3 from *P. berghei* (Pb), *P. falciparum* (Pf) and *P. vivax* (Pv), and that of yeast YCA1. Amino acid identity and similarity shared by at least five of the aligned sequences are shaded. Numbers in parentheses denote numbers of residues not shown. Asterisks marks the position of predicted catalytic dyad histidine and cysteine residues. Sequences are based on PlasmoDB gene IDs PB001074 (PbMC1), PB000485 (PbMC2), PB000564 (PbMC3), PF13_0289 (PfMC1), PF14_0363 (PfMC2), PF14_0160 (PfMC3), Pv114725 (PvMC1), Pv118575 (PvMC2) and Pv08564 (PvMC3).

Using BLAST searches of available *Plasmodium* genome sequences with the amino acid sequence of PfMC1 we identified orthologues in *P. berghei* and other *Plasmodium* species including *P. chabaudi*, *P. yoelii*, *P. knowlesi*, *P. vivax* and *P. gallinaceum*. Multiple alignment of the predicted PxMC1 proteins revealed conserved amino- and carboxy-terminal domains, separated by a region of variable length and sequence. These amino- and carboxy-terminal domains had, in Pfam and SMART searches, significant homologies with a C2 domain (a Ca^2+^-dependent membrane targeting module, Pfam identifier PF00168) and with the Peptidase_C14/Caspase domain (Pfam identifier PF00656), respectively (Fig. 1A). We also identified two paralogues of PxMC1 (named PxMC2 and PxMC3) in all *Plasmodium* species, each containing a region of significant homology to the Peptidase_C14/Caspase domain in Pfam and SMART searches. In contrast to the PxMC1 proteins however, sequence conservation in the much larger PxMC2 and PxMC3 proteins appeared to be limited to their C-terminal regions containing the putative caspase domains (Fig. 1A).

Multiple alignment of the predicted caspase domains of PxMC1, PxMC2 and PxMC3 from *P. berghei*, *P. falciparum* and *P. vivax*, along with a metacaspase from yeast (YCA1) [6] shed further light on their structures (Fig. 1B). Like PfMC1, all PxMC1 orthologues possess histidine and cysteine residues predicted to form the catalytic dyad in this family of proteases. It is interesting to note that PbMC1 has the predicted catalytic cysteine located one residue upstream of the consensus position for this amino acid, with a proline in the consensus position (Fig. 1B). The same is true for PcMC1 and PyMC1 (data not shown). In contrast, PvMC1 has two adjacent cysteines, at the consensus position and the preceding residue (Fig. 1B). The same is found in PkMC1 and PgMC1 (data not shown). Catalytic dyad histidine and cysteine residues are absent in the consensus positions in the PxMC2 and PxMC3 proteins (Fig. 1B). However, PfMC3 and PvMC3 have a cysteine at the preceding amino acid position, similar to the situation in PxMC1 of rodent malaria species.

### 3.2 Generation and molecular analyses of PbMC1 knockout parasites

**Fig. 2:**
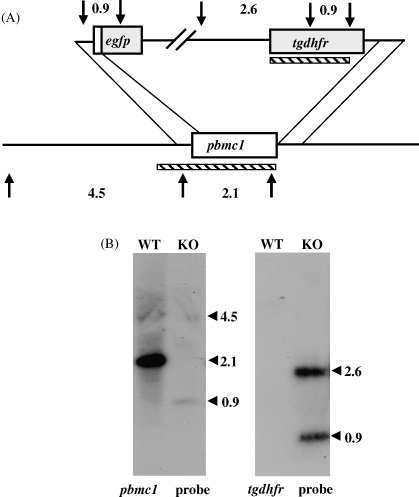
Construction of PbMC1-KO parasites. (A) Schematic diagram of the gene-disruption strategy. *Hin*cII sites are indicated by arrows, with predicted restriction fragment sizes given. Probes used in Southern blot are indicated by hatched bars. (B) Southern blot analysis of *Hin*cII-digested WT and PbMC1-KO parasite genomic DNA. Fragment sizes (in kb) are indicated.

Because PxMC1 proteins display the greatest sequence similarity with the consensus Peptidase_C14 domain and, of the three *Plasmodium* metacaspases described here, are therefore most likely to be active enzymes, we decided to investigate the expression of PbMC1 and its contribution to *P. berghei* parasite development. To this purpose we generated genetically modified parasites in which the PbMC1 coding sequence was removed and replaced with a reporter gene, enhanced green fluorescent protein (EGFP). In the strategy used, the 5′UTR and 3′UTR of the *pbmc1* gene allow for double homologous recombination [15,16], introducing the EGFP coding sequence downstream of the first 11 codons of PbMC1 (coding for MSLQMDKIYVK), as well as inserting a modified *T. gondii* dihydrofolate reductase/thymidylate synthase (*tgdhfr*/*ts*) gene cassette conferring resistance to the antimalarial drug pyrimethamine into the *pbmc1* locus (Fig. 2A).

After transfection of purified *P. berghei* schizonts [15] we readily obtained pyrimethamine-resistant parasites. Diagnostic PCR amplification across the predicted integration sites showed that correct integration of the *DHFR*/*TS* cassette into the *PbMC1* locus had occurred (data not shown). This was confirmed by assessing the integrity of a clonal population of the genetically modified parasite (named PbMC1-KO) by Southern blot analysis of *Hin*cII-digested genomic DNA. Accordingly, a probe corresponding to the 5′UTR plus PbMC1 coding region gave rise to expected bands of 4.5 and 2.1 kb in the parental wild-type (WT) parasites (Fig. 2B). In contrast, in the PbMC1-KO parasites expected bands of 4.5 and 0.9 kb were observed. The 0.9 kb fragment corresponds to 360 bp of the 5′UTR of *pbmc1*, its first 11 codons, plus 490 bp of the *egfp* gene (Fig. 2B). The 4.5 and 0.9 kb bands are more weakly labelled than the 2.1 kb band, because a smaller portion of the probe anneals to these fragments (Fig. 2A). A probe corresponding to the *tgdhfr*/*ts* gene gave rise to bands of 2.6 and 0.9 kb in the PbMC1-KO parasites, but no signal in the WT parasite sample as expected (Fig. 2B). These combined results are fully consistent with the structural removal of the PbMC1 coding sequence, and the integration of the reporter and selectable marker genes into the *pbmc1* locus.

### 3.3 PbMC1 expression and loss-of-function phenotype

**Fig. 3:**
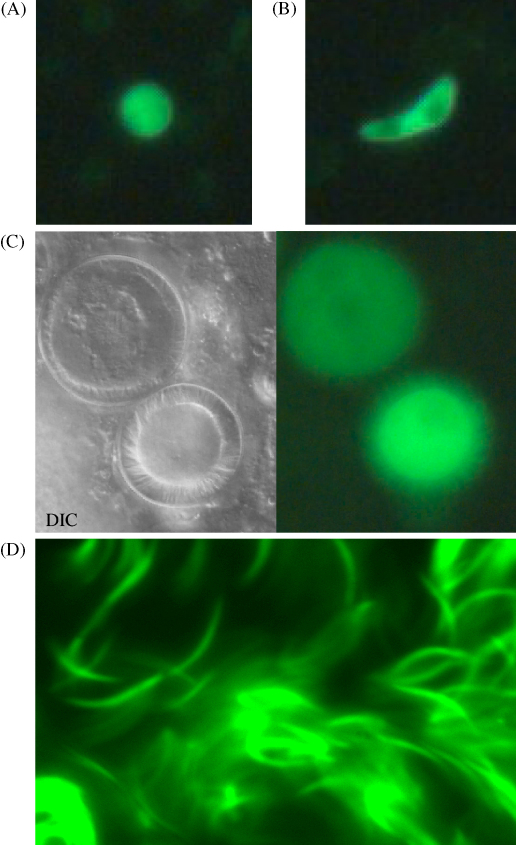
EGFP fluorescence in PbMC1-KO parasites. (A) Female gametocyte. (B) Ookinete. (C) Day 15 oocysts containing sporozoites. DIC, differential interference contrast. (D) Sporozoites inside an infected mosquito salivary gland.

PbMC1-KO parasites developed normally in mice and were morphologically indistinguishable from WT parasites in Giemsa-stained blood smears. Using UV microscopy EGFP fluorescence was readily observed in a subpopulation of parasitized erythrocytes. Upon gametocyte activation (by placing the infected blood in ookinete medium) the large majority (>90%) of fluorescent parasites emerged from the host cell (clearly visible by the rounding up of the parasite) by 15 min, typical of gametogenesis. The number of remaining intracellular fluorescent parasites was considerably lower than the percentage of trophozoites present in the sample as assessed by Giemsa staining. These are therefore likely to constitute immature gametocytes, or gametocytes that had failed to emerge. We were further able to distinguish between male and female gametocytes by observing exflagellation (*i.e*. the release of male gametes); this involved gametocytes that were not green. From these combined observations we conclude that the *pbmc1* gene is expressed only in female gametocytes (Fig. 3A). As far as we could ascertain all females were positive and all males negative. Normal differentiation of gametocytes into ookinetes both *in vitro* and *in vivo* was observed. These ookinetes again displayed bright EGFP-based fluorescence under UV light (Fig. 3B). WT and PbMC1-KO parasite-infected *A. stephensi* mosquitoes developed comparable numbers of oocysts, indicating that the PbIMC1a-KO ookinetes are capable of normal midgut invasion and subsequent ookinete-to-oocyst transition on the hemocoel side of the midgut wall. Examination of oocysts on the midgut wall by UV microscopy displayed EGFP fluorescence in oocysts and the sporozoites contained within (Fig. 3C). Fluorescent sporozoites were observed in the mosquito salivary glands (Fig. 3D), which were infectious to mice upon infected mosquito bite. These combined results show that the *pbmc1* gene is expressed in female gametocytes and all downstream mosquito stages including sporozoites, but not in the asexual blood stages. The apparently normal development of the PbMC1-KO parasite both in the vertebrate and insect hosts shows that *pbmc1* is not an essential gene and may be functionally redundant.

### 3.4 Apoptosis in ookinetes

**Fig. 4:**
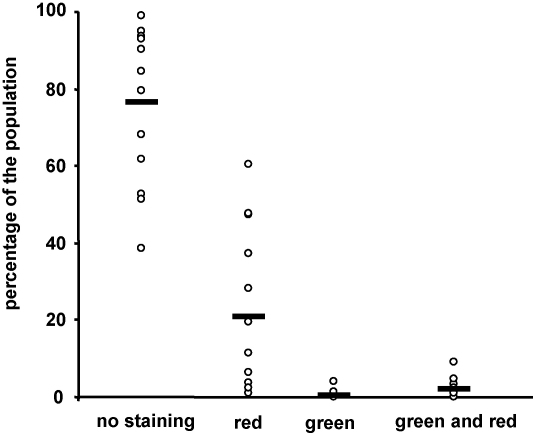
Diagram of proportion of Annexin V-FITC (green) and propidium iodide (red) labelled *in vitro* cultured *P. berghei* ookinetes. Circles shows values for individual experiments, bars represent average values across 13 experiments.

It has been reported that, in *P. berghei*, a large proportion of ookinetes undergo cell death, displaying typical apoptosis-like features such as PS translocation, nuclear condensation and DNA fragmentation [12]. As caspases are well known to play a central role in apoptosis, we wanted to assess the effect of PbMC1 knockout on this process. Initially, we assessed the level of apoptosis-like cell death in WT ookinetes. Annexin V conjugated to FITC (staining green), which specifically binds to PS, was used to assess the proportion of ookinetes displaying PS translocation to the outer leaflet of the cell membrane. Propidium iodide (staining red) was used simultaneously to assess plasma membrane integrity (cell viability). Across 13 experiments conducted we observed an average 77% viable ookinetes (no staining), while an average 21% appeared dead (red only staining) (Fig. 4). The average proportion of ookinetes displaying PS translocation (green staining) was less than 3%. Only 0.5% of ookinetes examined showed PS translocation and were propidium iodide negative (green only staining) (Fig. 4). In addition to this assay, we used acridine orange staining to look for nuclear condensation, and TUNEL to assess DNA fragmentation in *P. berghei* ookinetes, as described [12]. We found no evidence for either of these processes occurring.

Given the low numbers of positive cells in the above assays, we decided to look for the activation of caspases, a key event in caspase-dependent apoptosis, by using a fluorochrome-labelled inhibitor of caspases (CapaTag). In mammalian cells, these labelled inhibitors are permeant and covalently bind to the active centres of caspases with a 1:1 stoichiometry. In this assay, WT ookinetes displayed an average 3.8 ± 0.5% of positive cells (two experiments) after 21 h of culture, which, at 24 h had gone up to an average 14 ± 9% (four experiments). Under the same conditions, PbMC1-KO ookinetes showed an average 2.6 ± 0.3% positive cells (two experiments) at 21 h, and 13 ± 6% (five experiments) at 24 h of culture. We also used a green fluorescent *P. berghei* parasite with an intact *pbmc1* gene (PbGFPCON [17]), that displayed an average 3.8% and 9.0% of positive ookinetes at 21 and 24 h, respectively. Thus, knockout of PbMC1 does not appear to significantly affect the percentage of ookinetes that bind CaspaTag.

## 4 Discussion

In this paper we describe the generation of *P. berghei* parasites in which the caspase-like protein PbMC1 is replaced by the fluorescent reporter EGFP, allowing us to study both its expression profile and its loss-of-function phenotype using a single genetically modified parasite line. Our EGFP reporter data show the *pbmc1* gene to be transcribed, and probably translated, in female gametocytes and all downstream mosquito stages including sporozoites, but not in asexual blood stages. However, we cannot rule out that the *pbmc1* gene may be subject to translational repression. There are many examples of genes that are transcribed in the female gametocyte, but which have a translational block and are not translated until later in development, as was recently published [18]. Interestingly, *pbmc1* is not one of the genes identified by these authors, supporting a scenario of direct translation. Knockout of PbMC1 expression does not seem to adversely affect any stage of parasite development, at least *in vivo* in the mouse and mosquito, or in the culture systems used in this study. This was surprising because *pxmc1* is a highly conserved single copy *Plasmodium* gene, and because PxMC1 is predicted to possess consensus catalytic dyad residues and thus is likely to possess caspase activity. Although the two paralogues, PxMC2 and PxMC3, do not share the typical consensus catalytic dyad residues of the Peptidase_C14/Caspase family, it is possible that they constitute active zymogens by using alternate, functionally equivalent catalytic residues. Indeed, functional replacement between cysteine, serine or threonine residues has been reported for other cysteine and serine proteases [19–21]. If this is the case it is conceivable that one or both of the paralogues could carry out the role of PbMC1, hence leading to functional redundancy and resulting in an apparently normal mutant phenotype as observed. Interestingly, five metacaspase genes have been identified in *Trypanosoma brucei*, TbMCA1-TbMCA5, and two of these also lack a conserved histidine residue (replaced by a tyrosine, TbMCA1) and/or cysteine residue (replaced by a serine, TbMCA1 and TbMCA4) (reviewed in [22]). Whether any of these molecules are active enzymes remains to be determined.

It has been previously shown in plants and fungi [6,9] that metacaspases can be involved in the regulation or the execution of programmed cell death. Because an apoptosis-like mechanism of cell death was reported to take place in ookinetes of *P. berghei* [12] we hypothesized that PbMC1 could be involved in this phenomenon. Of three typical apoptotic markers: PS translocation, nuclear condensation and DNA fragmentation, only PS translocation gave positive results in our hands. PS translocation does also occur when cells die from accidental cell death (oncosis), in which case membrane integrity is lost concomitantly. Accordingly, we cannot truly determine whether ookinetes that in our assay stained doubly positive for both Annexin V and propidium iodide were in the later stages of apoptosis, or whether they died from an unrelated death mechanism. The fact that a subset of ookinetes stained positive for Annexin V suggests that the low numbers of positive cells observed are not a result of a reduced PS content in the parasite plasma membrane. Overall, the results based on these three apoptotic markers indicate that apoptosis-like death in ookinetes, in our hands, is low.

When we monitored for potential apoptotic ookinetes using CaspaTag, we obtained up to 14% positive cells. Surprisingly, no reduction of CaspaTag labelling was observed in PbMC1-KO parasites. Although PbMC1 is the most likely target of the CaspaTag inhibitor, it is possible that other targets of CaspaTag are present in ookinetes, which could mask any reduction in CaspaTag binding in the PbMC1-KO parasite. Clearly, the other two putative metacaspases are prime candidates. Indeed, *P. falciparum* transcriptome and proteome analyses indicate that PfMC2 is expressed in gametocytes and sporozoites [23–25]. Hence, its expression in other mosquito stages including ookinetes is likely. This scenario could also explain the normal development of the PbMC1 null mutant parasites by assuming a level of functional redundancy between the three *Plasmodium* metacaspases. Expression and knock out experiments with the other metacaspases are underway to test this hypothesis.

Caspase activation is one of the earliest events in apoptotic mammalian cells. Hence, early apoptotic cells have activated caspases, but display no changes in plasma membrane integrity [26]. It was therefore notable that no EGFP-based fluorescence was observed in CaspaTag positive ookinetes neither in the PbMC1-KO nor PbGFPCON parasite lines, indicating that these cells had lost plasma membrane integrity before CaspaTag labelling occurred. This is consistent with results from Al-Olayan et al. [12] who reported that their caspase inhibitor-positive ookinetes were propidium iodide permeable. Moreover, we found that pre-treatment with the unlabelled inhibitor failed to reduce subsequent binding of CaspaTag (data not shown). In mammalian cells this has been reported to reduce CaspaTag binding by over 90% [26]. We should thus consider the possibility that these caspase inhibitors may have different specificities in *Plasmodium* than they do in mammalian cells.

It is difficult to explain why in our hands the number of potentially apoptotic ookinetes detected was far below the numbers previously reported [12]. Despite numerous attempts we were unable to find conditions that led to increased numbers of ookinetes displaying apoptotic markers. We cannot rule out that small differences in the experimental set-ups might explain the conflicting findings. Alternately, the level of apoptosis detected in *P. berghei* ookinetes may depend on multiple factors that are still to be defined, for instance the exact conditions under which the gametocytes are generated in the mouse host. A not dissimilar discrepancy also exists for the reported apoptosis-like DNA fragmentation that occurs in blood stage *P. falciparum* parasites in response to chloroquine treatment [11], an observation that was not corroborated in a more recent study [27].
